# Surgical and radiological behavior of MRI-depictable mesh implants after TAPP repair: the IRONMAN study

**DOI:** 10.1007/s10029-019-02019-2

**Published:** 2019-07-31

**Authors:** M. Lechner, M. Meissnitzer, K. Borhanian, R. Bittner, R. Kaufmann, F. Mayer, T. Jäger, S. Mitterwallner, K. Emmanuel, R. Forstner

**Affiliations:** 1 Department of Surgery, Paracelsus Medical University, Müllner Hauptstraße 48, 5020 Salzburg, Austria; 2 Department of Radiology, Paracelsus Medical University, Müllner Hauptstraße 48, 5020 Salzburg, Austria

**Keywords:** MR visible, MRI, TAPP, Inguinal hernia, Mesh repair, Shrinkage

## Abstract

**Purpose:**

Knowledge of postoperative behavior of mesh implants used for hernia repair is generally limited to cases of recurrence, local complications or return to the previous operative field in other pathological conditions. Previous studies with MRI-visible mesh implants in different parts of the abdominal wall have led to variable findings with regard to mesh properties and mostly described a reduction in size over time with subsequently limited mesh overlap over hernia defects which could contribute to recurrence. We aimed to evaluate implant properties in a mechanically stable anatomical region after TAPP repair of primary unilateral inguinal hernias in men with clinical and MRI examinations 4 weeks and 1 year after surgery.

**Methods:**

From 11/2015 to 01/2019, 23 men with primary, unilateral, inguinal hernias underwent TAPP repair with iron particle-loaded, MRI-visible mesh implants in a prospective cohort study. In 16 patients the operative outcome could be evaluated 4 weeks and 12 months after surgery by clinical examination and MRI evaluation with regard to postoperative course, possible adverse outcomes and radiological findings related to implant behavior—namely MRI-identifiability, mesh dislocation or reduction in surface area.

**Results:**

All included patients had an uneventful postoperative clinical course. MRI after 4 weeks revealed one postoperative seroma, which resolved spontaneously. No recurrence was detected. Mesh implants could be accurately delineated in DIXON-IN studies and showed neither clinically nor statistically significant changes in size or position.

**Conclusion:**

4 weeks and 1 year after a standardized TAPP procedure the mesh implant used in this study showed no tendency towards dislocation or reduction in size in this anatomical position. Its MRI visibility allows accurate delineation during the postoperative course by experienced radiologists in appropriate MRI protocols. Larger patient series are desirable to further support these findings. Shrinkage of implants in the groin as a reason for early recurrence may be overestimated.

## Introduction

Despite the ever-increasing focus on postoperative chronic pain, recurrence rates remain a mainstay of the outcome evaluation in hernia surgery. Apart from the operating surgeon’s experience and accurate surgical technique, device properties, namely suspected shrinkage of the implanted mesh, are often accused of being the underlying cause of recurrence. Since most conventional implants cannot be delineated with sufficient precision in magnetic resonance (MRI) or computed tomography (CT) imaging, conclusions of in vivo implant behavior for a long time could only be drawn from intraoperative findings in case of re-operation for either recurrence or other reasons that led to patients’ return to the operating theatre. Both settings, of course, yield a high level of selection bias. This changed with the introduction of mesh implants loaded with iron particles that have proven to be visible in cross section imaging in various previous studies [1–3]. All of these aimed to evaluate presentability and postoperative changes in mesh size and surface area after implantation in either laparoscopic or open intraperitoneal onlay mesh (IPOM)-, retromuscular sublay-, or infra diaphragmatic onlay (for hiatal hernia augmentation)—position. Two studies [1, 3] focused on inguinal hernias. All trials to date include a limited number of patients and evaluate in vivo mesh behavior in very different anatomical positions [1, 3–6]. In addition, postoperative mesh size and surface were usually analyzed at different points in time. All these factors do not discredit the mentioned publications in any way but may lead to limited reproducibility of the obtained results. Some trials evaluate the changes in implant size and shape after deflation of the abdomen at the end of laparoscopic procedures and find relevant changes in effective mesh size with regard to the initially planned overlap over hernia defects [5]. Lessons from previous valuable efforts in this field of research were learned and it is the aim of this study to evaluate the proneness of the chosen mesh implant towards shrinkage in a setting that yields as little variability of potentially biasing factors as possible.

## Material and methods

### Surgical care

From 11/2015 to 01/2019 a prospective single-center cohort study was conducted, 23 Caucasian patients were recruited and underwent transabdominal preperitoneal plasty (TAPP) repair for primary, unilateral, inguinal hernias.

Inclusion criteria were age > 18 years, male gender, symptomatic unilateral inguinal hernia EHS (European Hernia Society) size I or II, informed consent, American Society of Anaesthesiologists (ASA) classification I or II status and willingness to participate in the Herniamed® Register.

**Fig. 1:**
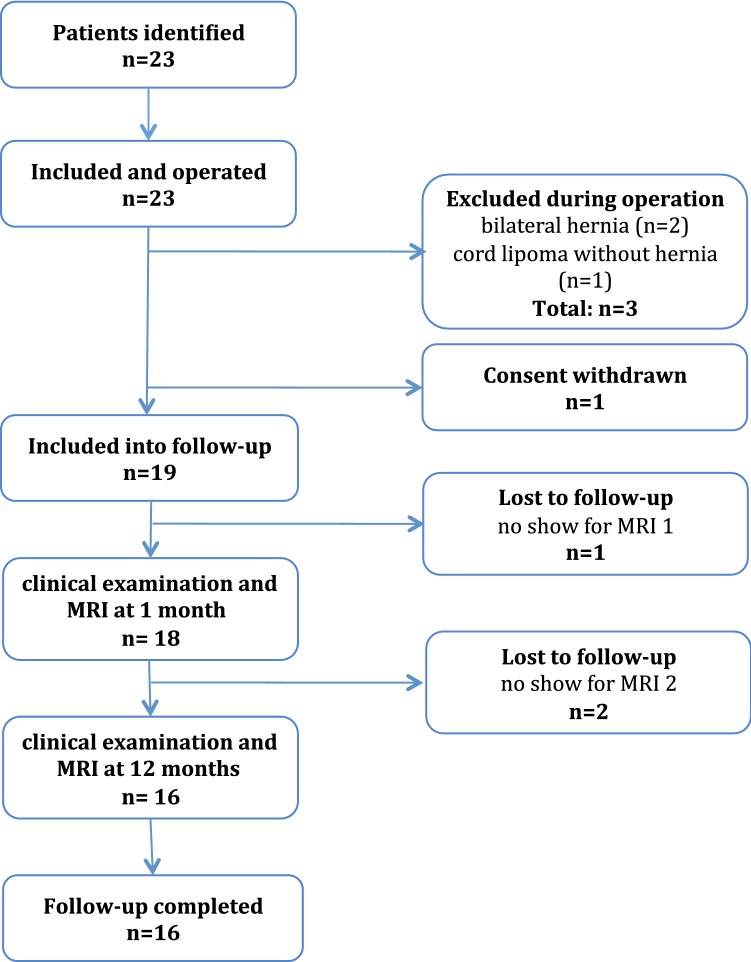
Flowchart of the study design

Exclusion criteria were female gender, emergency procedures, age < 18 years, scrotal or irreducible hernias, evidence of local inflammation or peritonitis, history of previous abdominal surgery or pelvic trauma, language barriers limiting understanding of the study-protocol or follow-up, presence of metallic implants (namely pace-makers, cochlea-implants and osteosynthetic devices) or tattoos prohibitive of safe magnetic resonance imaging (MRI), as well as general contraindications for MRI examinations. The flowchart of the study design is outlined in Fig. 1.

Primary endpoints were the evaluation of changes in mesh size, position or configuration between 4 weeks and 12 months after surgery. Secondary endpoints were the evaluation of mesh visibility with different MRI protocols as well as the analysis of clinical outcomes with regard to possible changes in mesh size or position. Preoperatively, all patients received a clinical examination in supine and erect position at rest and during Valsalva, as well as a grey-scale sonography of the groin, all carried out by one of the two operating surgeons.

Of the 23 operated patients, 4 were excluded from the study due to intra-operative findings of previously undiagnosed bilateral hernia (*n* = 2), sole presence of a spermatic-cord lipoma without hernia sac (*n* = 1) or postoperatively revoked consent (*n* = 1). Of the 19 remaining patients, 3 failed to complete the entire study protocol and were therefore declared lost to follow-up. Eventually, the study cohort comprised 16 patients. Demographic and hernia specific data are displayed in Table 1.

**Table 1:** Demographic and hernia specific data

Age (year)	Mean (SD)	50.8	(14.2)
Height (cm)	Mean (SD)	180.0	(7.4)
Weight (kg)	Median (range)	84.0	(60–94)
BMI (kg/m^2^)	Mean (SD)	25.0	(2.1)
Smoker	*n*	3	
ASA I	*n*	11	
ASA II	*n*	5	
VAS (preop)			
0	*n*	9	
1	*n*	2	
2	*n*	4	
3	*n*	1	
Site			
Lateral	*n*	12	
Medial	*n*	4	
Size (EHS)			
1	*n*	1	
2	*n*	15	

All participants were operated by two dedicated hernia surgeons according to the standardized surgical TAPP routine meticulously described by Bittner [7].

**Fig. 2:**
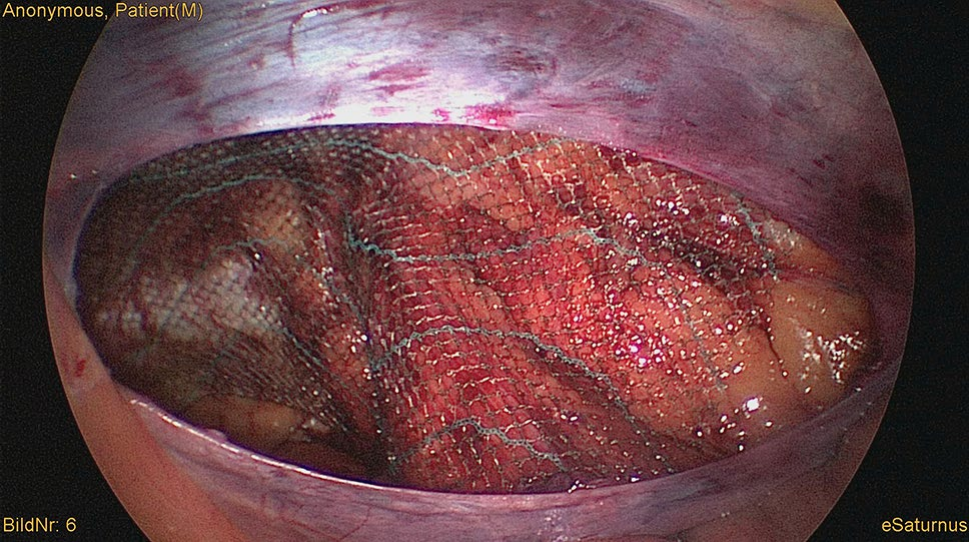
Intraoperative view of the groin with mesh implant in final position before closure of the peritoneum

All procedures were carried out with unaltered 10 × 15 cm, DynaMesh^®^-ENDOLAP visible, FEG Textiltechnik, Aachen, Germany implants. Mesh features include macro-porosity, as well as inclusion of iron particles, allowing MRI visualization. No drains were used and all implants remained without additional fixation (Fig. 2). Peritoneal closure was achieved by continuous barbed suture (Covidien™, V-loc 180^®^).

Postoperatively, patients were not subdued to any limitation of physical activity during or after hospital discharge. MRI scans were conducted 4 weeks and 1 year postoperatively and were analyzed by two independent radiology consultants. In addition, all participants received a standardized follow-up by questionnaire and telephone interview, according to the Herniamed® register’s protocol, as well as a clinical examination identical to the preoperative examination after each MRI. During the personal follow-up interview the groin regions were again examined clinically and possible symptoms at rest and during Valsalva’s maneuver were evaluated in erect and supine positions. For limitation of bias, both surgical and radiological investigators were blinded with regard to each others’ examination results.

## Radiological assessment

### Imaging technique

MRI exams at 1 month and 1 year after surgery were performed with a 3 T MRI scan (Philips Achieva, Best, The Netherlands). The following sequences were performed: transversal T2 Spair and transversal and coronal mFFE (each 4 mm slice thickness; scan duration 3:25, 2:52 and 3:35 min, respectively), multiplanar sDIXON-In and DIXON W (voxel size 1 × 1 × 2, scan duration: 1:24 min, FOV 220 as well as transversal T2 SSH (5 mm slice thickness, scan duration 30 s) at rest and during Valsalva.

### Imaging analysis

**Fig. 3 and 4:**
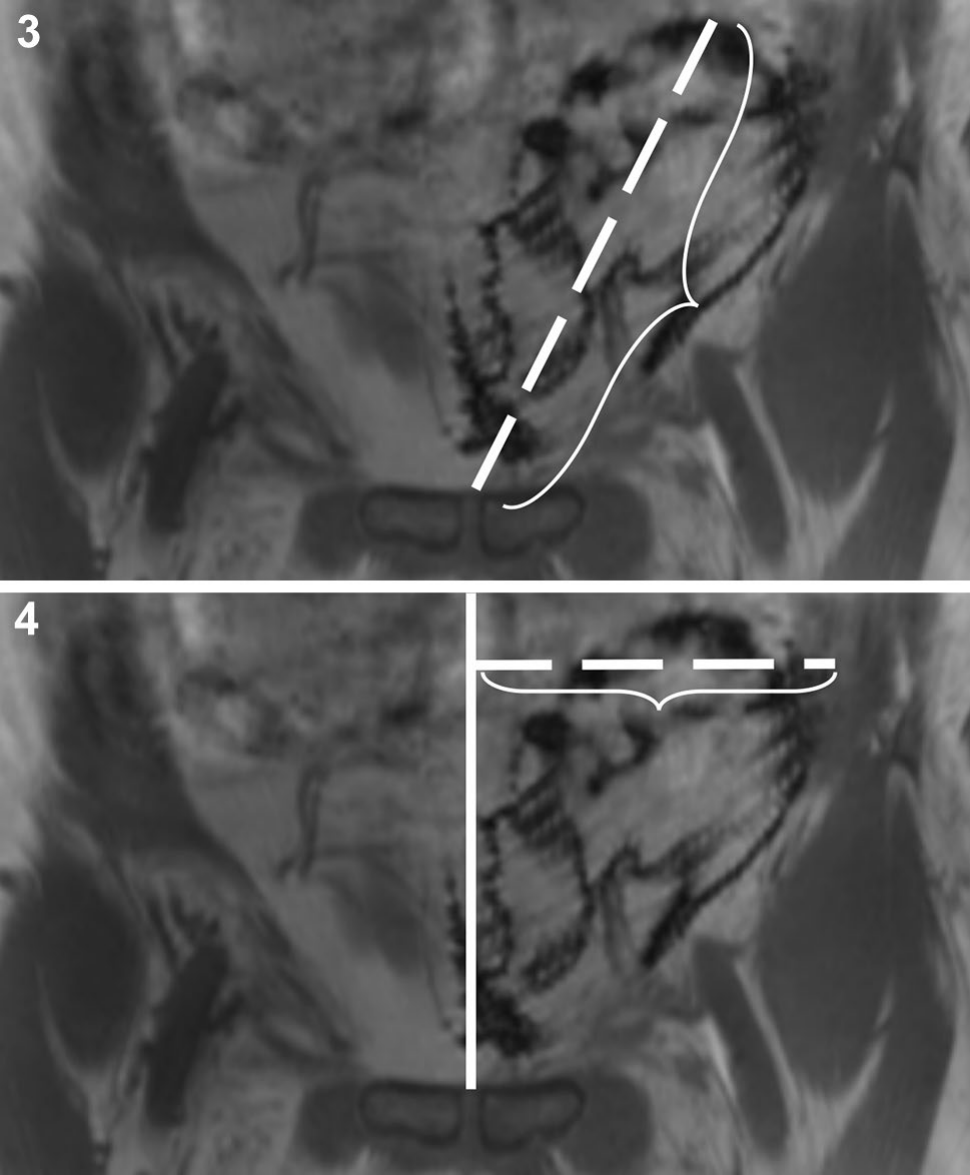
Movement of meshes was evaluated by measuring the maximum distances between cranial or lateral mesh margins and the pubic symphysis (**Fig. 3**) or the anatomical midline (**Fig. 4**), respectively. Measurements were taken on coronal images

Maximum diameters of the meshes were obtained in transaxial, sagittal, and longitudinal direction on multiplanar DIXON-In images at 4 weeks after surgery and after 1 year, respectively. In addition, measurements of the distances (in millimeters) of lateral and cranial mesh margins to defined landmarks, namely the anatomical midline (ML) and the symphysis (Sy) were taken at the two defined time points (Figs. 3 and 4). All images were analyzed by two senior radiologists with 8 and 20 years of experience in MRI. Analysis was performed independently by visual inspection on dedicated workstations and then a joint reading session was scheduled in which all images were evaluated and discussed. Consensus was reached on each qualitative and quantitative parameter. Qualitative reading included analysis of mesh visibility on four different types of sequences. Presence of local surgical site findings like hematoma, seroma, meshoma, recurrent hernia, mesh dislocation, changes in implant shape, size or position was evaluated. In addition, quantitative analysis was performed by measurement of defined diameters and distances. All measurements were performed on DIXON-in images as these were rated as providing the best resolution of mesh implants and surrounding anatomy by both radiologists in all patients. Visibility of the mesh on various sequences was evaluated and rated on consensus by the two radiologists on a four-scale visibility scoring system as either excellent (+++), good (++), moderate (+) or poor (–) (Table 2). Quantitative measurements included maximum diameters of the implants on both MRI exams for each patient in three planes. Furthermore, and to objectify the position of the mesh with regard to anatomic landmarks, maximum distances of the most cranial margin of the implant to the symphysis and the maximum distance of the most lateral margin of the mesh from the midline on coronal DIXON-W images were recorded.

**Table 2:** Four-scale visibility scoring system for mesh visibility in different MRI sequences

Sequence	DIXON-IN	mFFE	T2FS	T2 SSH
Rating	+++	++	–	+

**Fig. 5:**
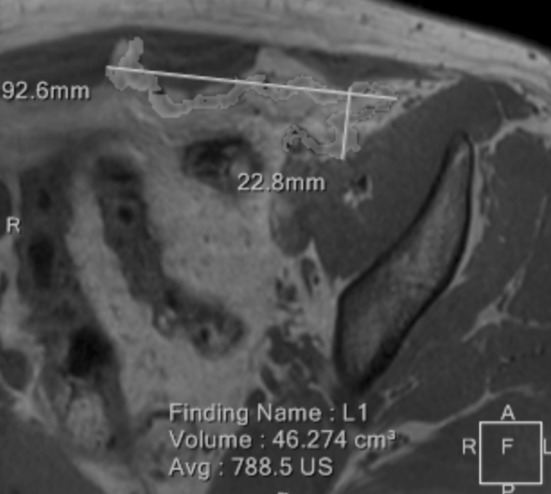
Volumetric measurements were performed semiautomatically on multiplanar DIXON-IN images

Eventually, volumetric measurements were added by a third radiologist by semiautomatic segmentation of the meshes on DIXON-W studies on a dedicated work station (Philips Intellispace, Best, The Netherlands) (Fig. 5). All results were entered into an Excel spreadsheet, mean values, standard deviations and changes in percent for each parameter with mean values and standard deviation calculated.

### Statistical analysis

We conducted a paired *t* test to determine whether, on average, there was a change in six measurements (mesh volume and five mesh distances) between 1 month and 12 months after inguinal hernia surgery by TAPP repair with iron-loaded mesh implants in 16 patients. Continuous variables were expressed as mean ± SD after checking normality of the differences with the Shapiro–Wilk-test. All tests are two-sided and a *p *value of less than 0.05 indicates a statistically significant difference. All statistical analyses in this report were performed using STATA (StataCorp. 2015. Stata Statistical Software: Release 14. College Station, TX: StataCorp LP).

## Results

### Surgical care

Over the course of 38 months 23 male patients with primary and unilateral inguinal hernias were recruited and met the rigorous selection criteria of the study. Mesh fixation or trimming of any type as well as drains was avoided per protocol. Intra-operatively three patients were excluded from further participation in the trial due to incidental findings of bilateral hernias (*n* = 2) or cord lipoma without an actual hernia (*n* = 1). One patient withdrew his previously given consent to participate in the trial immediately after the operation. Three more patients failed to present for radiological appointments at the defined time points and were subsequently declared lost to follow-up. Results of 16 patients with a median age of 54 years and a median BMI of 25.2 kg/m^2^ were eventually evaluated after 8 left-sided and 8 right-sided hernias had been repaired with a median procedure time of 44 min. Size and type of the hernias were determined according to the EHS classification: 12 lateral and 4 medial hernias were repaired. 15 hernias were rated EHS size 2, one was size 1. No intra-operative complications occurred and postoperatively no adverse outcomes like surgical-site infection, wound-healing disorder, hematoma, seroma, dysaesthesia or recurrence were clinically detected. Additional follow-up is carried out according to the standards of the Herniamed® registry.

### Radiological

**Fig. 6:**
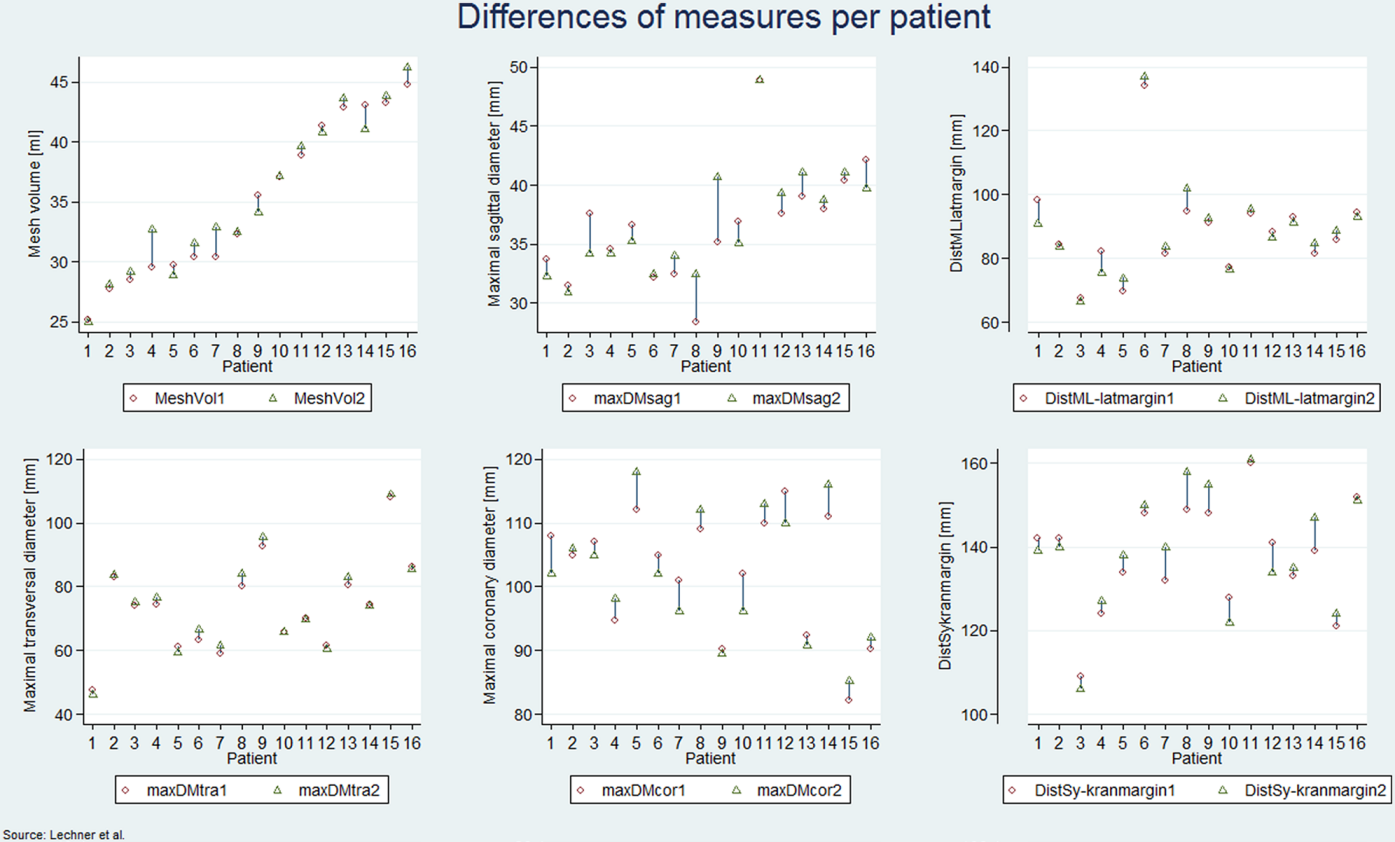
Differences in measurements per patient 1 and 12 months after surgery MeshVol, mesh volume; maxDMsag, maximal sagittal diameter; DistML-latmargin, distance midline and lateral mesh margin; maxDMtra, maximal transverse diameter; maxDMcor, maximal coronary diameter; DistSy-kranmargin, distance symphysis and cranial mesh margin

Results of the evaluation of images with regard to visibility of the mesh in various sequences are displayed in Table 2. Per consensus, implants were best visualized on DIXON-In images. Generally, the MRT readings showed an increase of the mean values except for the maximum coronary diameter where a mean decrease of 0.2 mm was observed (*p* = 0.85). The mean values of the reminder MRT readings showed an increase with a mean difference of 0.2–1.6 mm from the first to the twelfth month after surgery. In the analyzed sample no statistically significant changes of the six measured parameters between the first and the twelfth month after surgery were observed. In qualitative and quantitative imaging analysis no significant shrinkage or evidence of significant implant migration was found in MRI exams performed 4 weeks and 1 year after TAPP repair for the implant used in this study (Table 3, Fig. 6).

**Table 3:** Radiological measurements statistic

		Measurements						
		1 Month		12 Month		Difference		
		Mean	SD	Mean	SD	Mean	SD	*p*
Volume	[ml]	35.1	(6.6)	35.4	(6.4)	0.4	(1.3)	0.25
Diameter transverse	[mm]	73.9	(14.7)	74.8	(15.4)	0.9	(1.8)	0.07
Diameter sagittal	[mm]	36.6	(4.8)	36.9	(4.7)	0.3	(2.3)	0.58
Diameter coronary	[mm]	102.2	(9.5)	102.0	(10.0)	− 0.2	(4.0)	0.85
Maximum distance: midline—lateral mesh margin	[mm]	88.6	(15.0)	88.8	15.7	0.2	(3.8)	0.83
Maximum distance: symphysis—cranial mesh margin	[mm]	137.6	(13.0)	139.2	(14.7)	1.6	(5.0)	0.23

**Fig. 7:**
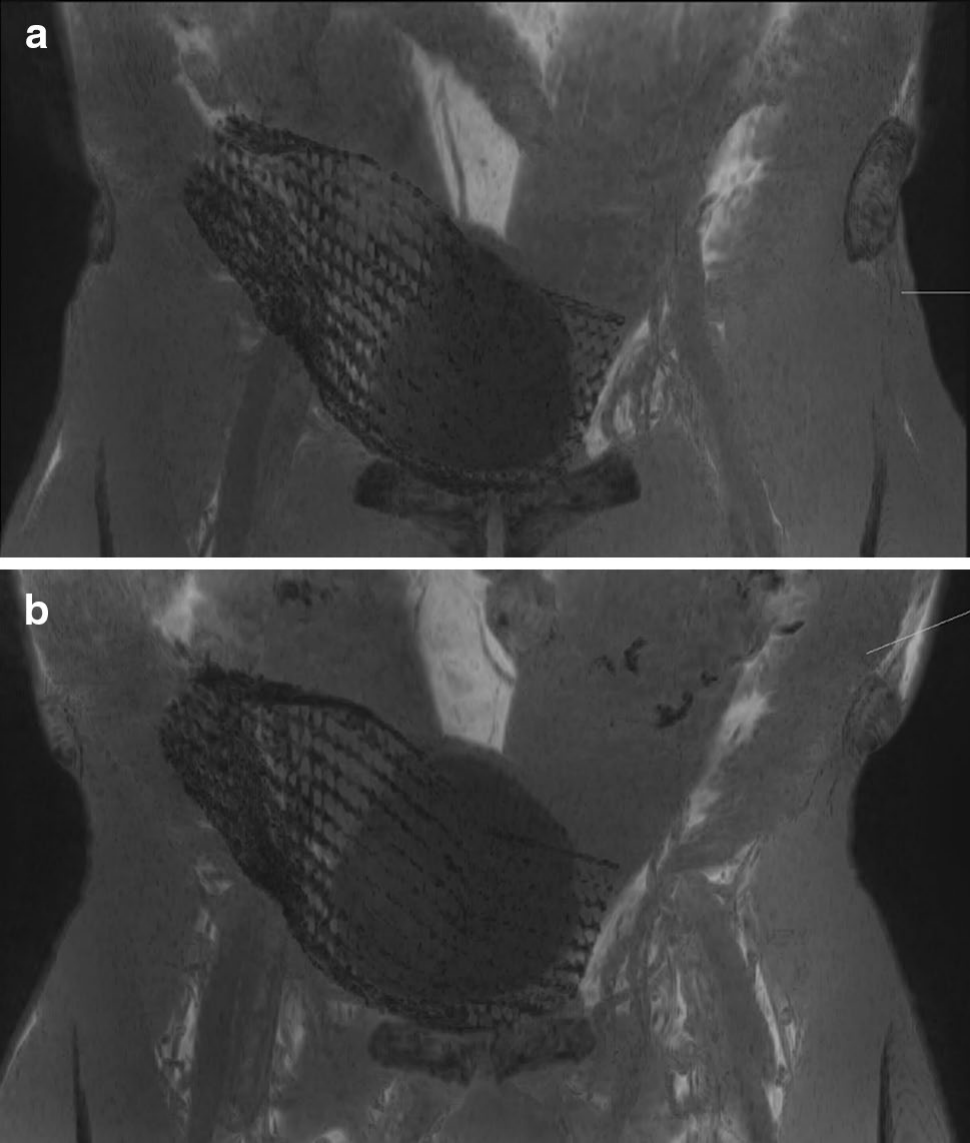
No significant change was found with regard to size and position of the implant between 1 month (**a**) and 1 year after surgery (**b**)

In the early postoperative MRI studies 1 postoperative seroma was detected. It resolved without intervention and was no longer present during the second study 1 year after surgery. No other complications, namely recurrence or formation of meshoma were detected at any point in time during the study period. Implants showed identical position and configuration after 4 weeks and after 1 year (Fig. 7a, b).

## Discussion

Changes in mesh size and configuration linked to in vivo properties of the implanted material, scarring processes or mesh migration have been discussed as potentially underlying reasons for local complications and recurrence after hernia surgery [1, 8]. Standard mesh implants, however, cannot be accurately depicted in MRI studies [9, 10]. Since computed tomography (CT) exposes patients to radiation and scheduled surgical re-evaluation of mesh position or shape at any point in time after implantation yields a relevant risk of morbidity, both are obviously not viable options in human trial participants. Limitations of studies involving the latter method of exploration in animals were found to be limited by several types of bias [2]. Iron-loaded mesh implants have repeatedly been described as a valuable solution to the problem [2, 3, 5, 11] since 2012. Yet, comparability of the published studies is limited by various factors: First, early experimental trials were carried out in agarose phantoms [1] or small animals [2] rather than in human patients. Second, implant materials are heterogeneous in the available literature [3, 4, 9]. Even after reduction of mesh materials to iron-loaded PVDF implants, differences in results are found due to their use in the repair of different types of hernias: results for primary, incisional, ventral, inguinal, parastomal, and diaphragmatic hernias are published. Authors used various types of mesh fixation including fibrin glue [6], interrupted [12] or continuous [3] sutures, as well as absorbable [4] and non-absorbable [9] tackers. In addition, implants were placed in a variety of positions relative to the layers of the abdominal wall: results for Lichtenstein, TAPP, laparoscopic or open IPOM, sublay, inlay and onlay repair are available. In the end outcomes were evaluated at a wide range of points in time, depending on the different study designs: several publications include immediate postoperative findings [1, 2] and compare them to implant properties prior to implantation [5] or to findings at varying postoperative set-points. Follow-up times range from 2 [3] to mostly 90 [1, 5, 6, 8] days with two longer observational periods of 6 and 13 months [4, 12]. The number of implants is generally small or very small with a median of 9 (range 2–124) and often evaluates results in both men and women [4, 6, 10]. Gender may not be a very important factor in other types of hernias but due to anatomical differences it could certainly influence the outcome in inguinal hernias.

With the present study we aim at shedding further light on the properties and in vivo behavior of the mesh implant chosen for the study in TAPP repair. The inguinal region was intentionally chosen as the area of interest since inguinal hernias are exceedingly common with a lifetime risk of 27% for men and 3% for women [13] and because laparoscopic inguinal hernia repair is increasingly carried out as routine procedure in many surgical departments. Furthermore, we aimed at selecting a surgical field that is subdued to respiratory changes during follow-up MRI examinations as little as possible. Compared to other areas of the abdominal wall like the ventral or diaphragmatic region, this should be the case in the groin with its partly osseous and therefore relatively stable wall. With 16 patients our study cohort was clearly larger than the median number of participants evaluated in other studies, but it is still relatively small. We aimed at compensating for that with rigorous patient selection and a small number of dedicated hernia surgeons who performed the TAPP repair according to a meticulously standardized surgical routine. For the same reason a late first set point for radiological baseline evaluation was chosen: 4 weeks after the procedure, immediate postoperative changes in the surgical field, like effects of the release of the capnoperitoneum or smaller hematoma could reasonably be expected to have subsided. The same was to be expected of residual carbon dioxide that has been known to partly impair mesh visibility on MRI [1]. In summary, the tight criteria for participation in the trial were defined to create the most homogenous cohort possible in view of the included number of patients.

With regard to outcomes no significant changes in mesh size or position were detected between 4 weeks after implantation and 1 year (Table 3, Fig. 6). The lateral aspect of the mesh adapted to the underlying anatomy, which results in a change of implant configuration towards a modified J-shape, compared to the intraoperative view (Fig. 2). These changes clearly occur in the early postoperative phase. They have also been observed by other authors [3] and are beyond the scope of this study. Most importantly, however, they remained unchanged throughout the study period. Overall minimum absolute changes in measurements were detected in the range of millimeters for one and the same patient at the two set points. These differences in dimension are put down to the measurements taken by the MRI readers and the semiautomatic nature of volume measurements that in ten patients revealed an increase of measured mesh volume, which in reality is not possible. None of the observed changes reached statistical or clinical significance. The somewhat larger differences in measurements between different patients of the cohort are explained by biometric differences between participants, which logically lead to different mesh configurations, depending on the individual anatomic surroundings. At the end of follow-up for this trial all implants covered the former hernia defects and showed no signs of local complications. Accordingly, no signs of recurrence were detected and no complaints were reported by the participants.

Limitations of the study may derive from the relatively small number of participants and the surprisingly long recruitment period for a very frequent surgical procedure. The latter is a result of the extensive exclusion criteria. Also the results obtained cannot be transferred to other areas of the abdominal wall, other types of implants or other surgical techniques. Given the vast number of inguinal hernias that are repaired in TAPP technique and without additional fixation of implants, the specific findings still affect everyday surgical routines and may be valuable for clinical decision making. Even though the follow-up period of this analysis is among the longest in literature for MRI-visible meshes, it is likely too short to deliver definitive answers about hernia recurrence and long-term mesh behavior over many years. For this reason patients are followed up according to the Herniamed® registry and their clinical courses will be monitored over the years to come. In case of reported or diagnosed complications, patients will be offered a re-evaluation by MRI. Strongpoints of the study are its prospective nature, the highly selected participants and the strict limitation to primary, unilateral inguinal hernias of EHS size I or II in a field unaltered by previous surgical procedures. The two comprehensive postoperative clinical examinations and the blinding of surgeons and radiologists with regard to each others’ results are further strong points of the study.

In conclusion, the mesh used for this analysis shows no statistically or clinically relevant changes in shape, position and configuration between 4 weeks and 12 months after implantation in TAPP technique. Differences between identical implants positioned in different patients appear more pronounced. All mesh implants could be outlined adequately in DIXON-In MRT imaging. Examination times with this protocol are short enough to provide patient comfort and to comply with busy radiological departments’ workload.

At the end of the follow-up period no complications had occurred. Early recurrence within 1 year after implantation of the device may therefore be caused by other factors rather than postulated mesh shrinkage. In the long view iron-loaded mesh implants may help to reduce the need for surgical exploration in case of local postoperative complications. With increasing knowledge of postoperative implant behavior, improved or even customized mesh design could soon be within reach for technically challenging or anatomically unusual hernia cases.
